# Types of Consciousness: The Diversity Problem

**DOI:** 10.3389/fnsys.2021.747797

**Published:** 2021-11-22

**Authors:** Carlos Montemayor

**Affiliations:** Department of Philosophy, San Francisco State University, San Francisco, CA, United States

**Keywords:** consciousness, attention, language, consciousness and agency, consciousness and attention

## Abstract

Consciousness research has a cognitive-diversity problem. Any view that holds that attention is either necessary for consciousness or that attention precedes conscious awareness confronts the difficulty that the theoretical categorization of attention is as diverse as the categorization of intelligent cognition, but consciousness is typically referred to as a single and unified capacity. On the one hand, we have a multiplicity of kinds of attention. On the other hand, we use a monolithic “phenomenal” notion of consciousness to define the dependency of consciousness on all these diverse kinds of attention. Since attention is defined in terms of a diverse variety of functions, a lot more needs to be said with respect to the claim that attention is either necessary for consciousness or that attentional processing precedes conscious awareness. Is this dependency based on the diverse cognitive functions of attention? If so, why conceive of consciousness as a single informationally unified cognitive capacity? What does the multiplicity of kinds of attention entail for consciousness research? This is the “diversity problem.” This article argues that consciousness should be also considered as a diverse set of capacities, based on the diversity of attention. While we have the intuition that consciousness is a unified perspective, the article shows that consistency demands this diverse approach. Since research on attention distinguishes a wide range of functions and levels of cognitive processing, the dependency of consciousness on attention entails diverse conscious capacities and diverse types of awareness beyond the distinctions between being awake, dreaming, and being minimally conscious.

## Introduction

### The Contrast Between Attention and Consciousness Research

Consciousness research has a cognitive diversity problem. Any view that holds that attention is either necessary for consciousness or that attention precedes conscious awareness must explain how exactly the unified capacity for conscious awareness depends on the great variety of types of attention that have been empirically verified. In fact, the theoretical categorization of attention is as diverse as the categorization of intelligent cognition. From object-based and feature-based perceptual attention, to voluntary and introspective attention, intelligent cognition is conceptualized and studied in multiple ways through diverse attention routines, across species and within species, as well as across sensorial modalities. These diverse forms of attention range from early sensorial processing to emotional and conceptual information. On the one hand, we have a multiplicity of kinds of attention. On the other hand, we use a monolithic “phenomenal” notion of consciousness to define the dependency of consciousness on the diversity of attention routines. Since attention is defined in terms of a diverse variety of functions, a lot more needs to be said about the nature of the necessity of attention for conscious awareness. I shall call this difficulty the “diversity problem.”

Briefly stated, the argument for diversity is as follows: 1. Phenomenal consciousness depends on attention for its informational content. This includes semantic contents, informational formats, as well as explicit and implicit access to information. 2. Attention is quite diverse in functions, salience or relevance structures, and informational contents across modalities and across species. 3. The variety of types of attention, given (1) should entail at least some diversity in types of phenomenal consciousness. Therefore, there must be various types of phenomenal consciousness. However, and contrary to this argument, phenomenal consciousness is *a priori* assumed to be a highly unified phenomenon, captured by the expression “what it is like for a subject to experience a content.” Since the argument in favor of diversity is not only plausible but also grounded on empirical evidence, this *a priori* assumption must be abandoned. Accordingly, the argument for diversity is not meant to be an instance of a general *a priori* argument to the effect that unity cannot emerge from diversity. Rather, the argument is meant to be specifically based upon the relation between phenomenal consciousness and attention. We can certainly conceive of something monolithic and unified emerging form a diversity of different elements. But the empirical evidence and the nature of information processing demonstrate that consciousness cannot be a monolithic information phenomenon, given that it depends on attention. Presented as a problem, we can ask: how can consciousness depend completely on attention and be monolithic or deeply unified given the diverse kinds of attention psychologists regularly study?

An immediate objection to the way in which this problem is formulated is that there are standard distinctions between types of consciousness, particularly in the philosophical literature, that seem to undermine the diversity problem. For instance, philosophers distinguish between access and phenomenal consciousness, levels of consciousness, creature and state consciousness, transitive and intransitive consciousness, as well as various types of self-consciousness (see Van Gulick, 2018). Many of these distinctions concern subjectivity, the first-person perspective, and the contents of conscious states. Thus, it seems futile to present a diversity problem based on considerations regarding attention since there are many diverse kinds of consciousness discussed in the philosophical literature^1^. This initial objection is erroneous, however, for a couple of reasons, both of which concern a misunderstanding of the diversity problem.

First, none of the distinctions above apply *exclusively* to phenomenal consciousness, which is the main topic of this article. In fact, Block’s (1995) distinction between phenomenal and access consciousness proposes that access consciousness need not have any specific phenomenology (or what it is like to experience it). This is the reason why some authors have proposed that access consciousness is best understood as an epistemically rich kind of attention (Montemayor and Haladjian, 2015; Stoljar, 2019). The other distinctions do not postulate anything specific about the diversity of phenomenal consciousness. The difference between creature and state consciousness concerns types of conscious states in contrast to the unique subjective perspective that is indexed to a creature or organism. Thus, the variety concerns organisms and different possible conscious states, rather than a variety of kinds of phenomenal consciousness. Transitive and intransitive consciousness similarly assumes distinctions concerning what it is like and the contents of conscious states but nothing specific about various kinds of phenomenal consciousness—the diversity here comes from contents and the associated experiences indexed to subjects. The self is another important source of variation, but again there is nothing specific here about phenomenal consciousness or its specific relation to the diversity of attention.

Thus, the diversity contained in these distinctions do not necessarily entail a diversity within phenomenal consciousness. However, and more important, even if these distinctions entailed specific kinds of diverse forms of phenomenal consciousness, there is no extant study of them *in relation to their dependence on types of attention*, which is the main topic of this article. Addressing this lacuna in the current literature on consciousness, both in philosophy and psychology, suffices to justify the present inquiry, even under the assumption that we have a clear understanding of the diverse kinds of phenomenal consciousness—which we do not really have, as the considerations just mentioned show.

Second, a more decisive and substantial response to this objection is that the diversity problem is a fundamental difficulty with respect to the relation between phenomenal consciousness and attention, which needs to be addressed independently of the multiple conceptualizations defended by philosophers. In other words, the diversity problem is an empirical issue that needs to be investigated through scientific methods. To do this properly, and in accordance with the scientific evidence, various kinds of phenomenal consciousness across species must be studied and specified in terms of attention. No conceptual maneuvering is going to help here. The diversity problem requires the investigation of various forms of phenomenal consciousness that presumably depend on the diverse, well-known, and empirically verified forms of attention.

It is relevant to note that the view that phenomenal consciousness (henceforth “consciousness”) and attention are identical does not solve the diversity problem and that in fact, it creates extra problems. If consciousness is the same as attention, then the multiplicity of various types of attention needs to be understood as various types of consciousness, but there is no theory that is committed to having as many kinds of consciousness as there are kinds of attention, for good reasons. Attention is a functionally defined selective capacity and a cognitive skill that delivers responses to contextual inputs. But on a standard, albeit not universally accepted understanding of consciousness, consciousness cannot be defined functionally (Chalmers, 1996). Even if one rejects, however, the view that consciousness defies functionalism, the claim that consciousness is identical with attention remains problematic because according to any account of consciousness, the diversity problem must be understood in terms of a difference in *information*.

The consequences of an informational difference between consciousness and attention, regardless of what notion of information one favors, are twofold. First, this distinction demarcates conscious from unconscious information, independently of considerations about semantic contents or metaphysical selves, which shows why the philosophical distinctions above are orthogonal to the topic at hand. Second, the diversity problem entails that various types of attention will contribute different kinds of information—an issue that this article addresses at length below. A dilemma presents itself: either consciousness is identical to attention or it is not. If it is, then there is no informational difference between consciousness and attention. If it is not, then consciousness is a monolithic phenomenon with a different, yet mysterious, informational and non-functional purpose. On a strong reading of the second horn of the dilemma, consciousness is monolithic because it cannot be studied with standard physical accounts of information (i.e., the hard problem). On a weaker and more empirically grounded reading, some types of attention, such as exogenous attention, might be necessary but not sufficient for consciousness (Chica and Bartolomeo, 2012), which implies informational distinctiveness but not monolithic unity. Thus, the diversity problem cannot be easily eliminated simply by identifying consciousness with attention. An account of conscious information must be provided, and this leads to either a denial of the uniqueness of consciousness or to the postulation of its informational and yet non-functional nature, which presents the problem of defining information in a non-functional manner.

Moreover, the identity view also generates the additional and quite considerable difficulty of eliminating the key notion of *unconscious attention*, which according to a vast number of studies, has been extensively confirmed at various levels of cognition (see for instance Dehaene, 2014). Unconscious attention does not seem to be an oddity of the human mind. On the contrary, it seems to be central for the proper functioning of human, and quite likely, animal cognition. Thus, saying that attention and consciousness are identical does not solve the diversity problem and it creates more difficulties that contravene the extant scientific evidence. Because of these difficulties, I will focus on the more accepted and empirically verified view that attention and consciousness are different cognitive processes. The diversity problem is challenging enough on a dissociation account of consciousness and attention. This article focuses on the question of how a dissociation account can be useful in addressing the diversity problem.

Diversity is not a problem either for attention or cognition—it seems to be a problem only for our peculiar understanding of consciousness. Similar to attention, diversity is an *advantage* with respect to intelligence. Generally intelligent organisms with capacities to make decisions and solve multiple problems in a variety of environments depend on such diversity. A broad categorization of these diverse capacities includes emotional, conceptual, communicative, navigational, as well as various capacities for learning, planning, and thinking. While these skills are integrated in an intelligent organism and there is significant overlap and cooperation between these skills in solving various tasks, there is clearly a wide variety of them, none reducible to the other. Diversity, therefore, only becomes a problem when confronted with the unity of consciousness and the related “hard problem” of consciousness. An initial step towards overcoming this problem is by challenging the intuitive appeal of the monolithic view of consciousness. There are other reasons to do this, which are independent of the diversity problem. For example, there is no empirical reason to think that there is only one kind of consciousness, within the human species or across species. All the “introspective evidence” about what it is like to experience contents is either inconclusive or based on self-assessments that might also be quite diverse (Sytsma and Machery, 2010) and even culturally dependent.

A philosophically inclined reader may argue that one should assume the unity of consciousness, based on our intuitions, until good reasons are provided against it. This is exactly what the diversity problem provides: good reasons to challenge the unity of consciousness on the basis of abundant evidence concerning its dependence on attention. The unity of consciousness is based on our intuitions, but the variety of kinds of attention is not—it has been verified extensively. So besides the evidence just mentioned concerning the cultural dependence of our intuitions, we have positive scientific evidence to doubt that consciousness is unified, given its dependence on the quite diverse kinds of attention.

In addition, in the phenomenological accounts of consciousness it is well understood that consciousness is quite diverse in *phenomenology*. Visual awareness is quite unlike auditory awareness, even when they are about the same contents. Emotional experiences are different from the experience of reasoning, and so on. Still, philosophers and scientists insist that there is a single kind of consciousness with specific and unique neural correlates in the brain. This is in sharp contrast with the correlates of attention and cognition or emotion, which are understood to be distributed in different areas of the brain—the diversity of attention and cognition is reflected in the diversity of anatomy and function. The concluding part of the article argues that even the notion of the first-person perspective may be understood as a diversity of agencies. The self, however, might be the most important source of unification of all the diverse kinds of attention and consciousness that depend on attention skills. Examining this possibility is beyond the scope of this article.

Conscious awareness may also vary during the life of an organism. It may be something entirely different to have the awareness of a child than to have the awareness of an adult (Gopnik, 2009). Just like attention and cognition morph into new and more integrated kinds of skills during cognitive maturation, so might consciousness development produce different types of awareness and skills that depend on metabolic stages of maturation. There could be diversity based on the distinction between top-down and bottom-up attention. For instance, the phenomenology of color illusions may depend upon low-level effects, while the phi-phenomenon could depend on higher-level processing concerning objects and shapes^2^. Yet another source of variation is the format of conscious contents and their valence. Some contents are metrically structured and analog, while others are conceptually structured or symbolic, and to that extent “digital.” Some are more intense than others; some we avoid and others we pursue. These issues are explored in what follows. The main point is that there are different styles of cognition, and this fact should have repercussions for consciousness research. Diversity should not be a problem, but rather an advantage.

However, even if one grants that there are multiple kinds of consciousness, the thorny question of the dependence of consciousness on attention persists. If attention is doing all the informational and cognitive work, then why do we need the category of “consciousness”? Since there is abundant empirical support for the claim that attention is necessary for consciousness and that attention comes in so many varieties, it is peculiar that the notion of a unified phenomenon called “consciousness” remains so influential. While it would be unproductive and unjustified to suggest that there are as many types of consciousness as there are types of attention—consider the fact that there are various types of unconscious attention—the presupposition that there is only one kind of phenomenal consciousness may be in fact an obstacle for scientific investigations, given that consciousness depends on attention.

The dependence of consciousness on attention, therefore, must be clarified by first addressing the diversity problem. The present proposal is to analyze two kinds of consciousness for which there is enough agreement in the literature, namely consciousness as subjective experience and consciousness as subject-level access to information, in terms of two broad categories of attention—conscious and not necessarily conscious. Further distinctions can be based on this broad categorization. Elucidating these distinct types of consciousness in this way could then help identify more specific neural correlates of consciousness, which will certainly help with the project of identifying the precise relation between consciousness on attention. It may be that they are totally different processes (see Montemayor and Haladjian, 2015, for discussion and review). But there is no way to tell prior to having a clear approach to the diversity problem, which presents obstacles to the science of consciousness. The next section examines how this problem might be an obstacle, more specifically, to the neuroscience of consciousness.

### The Dichotomous Science Concerning the Neural Correlates of Consciousness

The neural correlates of consciousness (NCC) can also be interpreted in terms of the distinction between awareness as either experience or access to information. This is one of the most plausible ways of interpreting the difference between cortical and non-cortical theories of consciousness, or alternatively, between consciousness and integrated attention. The key and shared assumption of all theories about the NCC is that consciousness is a single kind of phenomenon that can be identified with a single threshold of activation, in contrast to the multiple types of cognitive access and attention, spread across different areas of the brain. Thus, the assumption is that consciousness must have a unique neural instantiation or signature, distinct from all the various attention networks and neural correlates (sensory-dependent, object-based, spatial, voluntary, involuntary, etc.).

More specifically, this dichotomy can be stated anatomically, as the contrast between cortical and non-cortical theories of consciousness. Although they disagree about the location and nature of the NCC, all cortical theories propose that the prefrontal cortex, the sensory cortex, or a network involving cortical regions are uniquely responsible for conscious processing. The dominant views on the NCC are cortical, and they can be distinguished in this tripartite way at the anatomical level. The Global Neuronal Workspace Theory (GNWT), along with other “broadcast” theories, assume that extensive regions of the cortex must necessarily be activated to reach a threshold at which information becomes conscious (Baars, 2005; Dehaene, 2014). Higher Order Theories (HOT) presuppose a second layer of processing that associates contents with subjectivity, and which very likely involves the prefrontal cortex (Carruthers, 2000; Rosenthal and Weisberg, 2008). Local recurrent theories assume that awareness may be achieved by recursive processes in the perceptual regions of the cortex, and that such activation suffices for consciousness without the intervention of more extensive prefrontal regions or global neuronal networks (Lamme, 2003). According to this recurrent-processing approach, extensive activations might be required for access to information, rather than consciousness.

These cortical views are incompatible with evidence suggesting that the cortex is neither necessary nor sufficient for consciousness (Merker, 2007). Non-cortical views can be distinguished into two subgroups: thalamic and brainstem views. According to thalamic views, thalamic activations are at least necessary for conscious awareness, and might be even sufficient. In order to simplify the presentation of these views, I shall interpret them as claiming that the thalamic regions are necessary for conscious awareness. The absence of such activations in the thalamic network entails unconsciousness. In fact, many of the findings in support of this view are based on lesions or malfunction, and the study of coma states and minimal consciousness for clinical diagnoses.

Brainstem views propose an even more radical approach to the NCC. Based on the arousal-dependent motivational and emotional factors that accompany conscious experiences, these theories associate conscious processing with basic metabolic functions, such as homeostatic processes (Solms, 2021) or basic arousal (Merker, 2007). The key difference between brainstem views (particularly Solms’s) and other theories, is their emphasis on how metabolic processing imposes a *valence structure on conscious awareness*—conscious valence establishes the basic distinction between what “feels” good and bad. A consciousness-diversity approach could make cortical and non-cortical views compatible with one another, instead of our current conception of them as essentially rival approaches. Diverse conscious capacities can be structured both in terms of valence and semantic content or access to information. This entails a *diversity of formats*, as I explain in the next section.

Crucially, recent empirical evidence suggests that a hybrid and more conciliatory approach is needed. Diverse forms and stages of conscious information processing are integrated in distinct networks of the brain, with different functions and formats for encoding information. Findings show that the thalamus plays a fundamental role in conscious information processing, and that cortical activations are also important. The main limitation of current scientific approaches to consciousness is that, in spite of the diversity in approaches, there is no diversity in the target of study—there are many theories, relying on different methodologies and emphasizing various aspects of consciousness, all of them equally important, but they all assume that the object of study is not as diverse as the theoretical proposals. They all demarcate a sharp boundary between conscious and unconscious processing—a dichotomy between two types of information in the brain. Many findings on a wide range of psychological phenomena show that phenomenal experience cannot be explained by having specific contents because typically conscious contents can be processed unconsciously, as in cases of blindsight or in priming experiments (Vosgerau et al., 2008). The diversity of attention and cognition is in sharp contrast to this approach. It is in this sense that the contemporary science of consciousness is dichotomous: for all theories, cognitive processing is either conscious or unconscious, and this distinction is based on a single threshold of activation or a single kind of cognitive processing.

Partly because of the diversity of attention, a new scientific account of consciousness that is based on diverse processes will require an exploration of the degree of dissociation between consciousness and attention, including their possible mutual dependence in light of their evolution (Haladjian and Montemayor, 2015; Montemayor and Haladjian, 2015). It may be that some forms of consciousness are completely independent and irreducible to attention, while others are almost fully determined by specific forms of attention. The extreme options of complete dissociation and identity are unlikely, but should also be investigated. In any case, studying consciousness in relation to attention according to their degrees of dissociation may help unify the extant theoretical approaches to consciousness.

With respect to the integration of the available empirical evidence on consciousness and attention, an example of how different NCC could contribute to stages of conscious processing is the research on the thalamus and its projections to the brainstem and the cortex. Cortical theories underestimate the role of brain stem and thalamic processes in engendering consciousness. There is new evidence for the core idea that the thalamus is a gateway for producing consciousness, which dates back to the pioneering work of Crick and Koch (2003). The evidence shows that consciousness cannot be reduced to activations that depend exclusively on cortical networks and that the upper brain stem is fundamental. The thalamus seems to be necessary for consciousness as the central gateway enabling conscious processing. Its neural connections to the brainstem and horizontal ones with thalamico-cortical areas include top-down paths from the prefrontal cortex. Deep brain stimulation of the central lateral thalamus (CL) in anesthetized or sleeping macaques had the effect of waking them up (Redinbaugh et al., 2020). Redinbaugh et al. (2020) additionally describe that consciousness depends on “large-scale” thalamico-cortical and cortico-cortical interactions.

In addition, Stehberg et al. (2001) identified shared circuits for visceral signal processing and attention in the thalamus. Of particular importance to a hybrid approach regarding the NCC, Halassa and colleagues (Wimmer et al., 2015; Wells et al., 2016; Nakajima et al., 2019) demonstrated the central role of the thalamic reticular nucleus in the regulation of attention: in an attention task rats had to focus either on the acoustic stimulus or the visual stimulus presented at the same time in order to receive a reward. The underlying neural processes were found to involve bidirectional interactions between the thalamic reticular nucleus and the neocortex. Thalamic processes activated the neocortical processes, especially prefrontal cortex. Moreover, thalamic activations within a single pyramidal cell functioned as gateway modulators. If the thalamic gate is blocked, then the lower part of the pyramidal cell enables the processing of features activated by the stimuli, but these features are processed unconsciously and out of context (Aru et al., 2020).

In sum, although consciousness is conceived as a single and binary phenomenon (i.e., either subjects are conscious or unconscious) the dynamics between consciousness and attention reveal that various forms of awareness may play different roles, with potentially different thresholds of activations, arousal levels, and valence. In particular, recent findings about the NCC suggest that consciousness may depend on cortical regions for processing contents, but that it may also fundamentally depend on non-cortical regions for other key aspects of awareness, such as the visceral valence that characterizes emotion regulation and arousal. Thus, contemporary approaches to consciousness may not be as incompatible with one another as they might initially seem. Processing features and semantic contents may depend on areas that rely mostly on attention, and might in principle be dissociated from consciousness (Dehaene et al., 1998). Basic activations for emotion regulation and arousal might be operative in the absence of content processing. However, once content processing is integrated with basic conscious processing in non-cortical regions, a different and more flexible type of consciousness for learning and long-term memory emerges. The implications of the neuroscience of consciousness for the diversity of consciousness are clear. Arousal and valence play an important role in basic forms of conscious awareness associated with energy regulation and homeostatic processes. Further processing of these activations in the cortex gives rise to a more stable and long-term type of awareness. This diversity entails various forms of conscious formats for encoding information.

### A Diversity of Formats

Consciousness may be diverse not only with respect to its functions and NCC, but also with respect to how it encodes and integrates information through differences in information-formatting. A “format” is a type of informational system that organizes information in order to allow its storage and retrieval. This rather generic definition is sufficient to capture the kind of diversity of conscious awareness that depends on how information is encoded. For example, information about numbers can be formatted by discrete representations, such as tally marks, Arabic numerals, or Roman numerals. Depending on the task, some are better than others, although the Arabic decimal system seems to be the superior notation. Numbers can also be formatted by continuous representations, such as lines (see below the distinction between analog and digital formats). Lines are better at encoding information about real numbers. Similarly, information about objects, properties and contents in general can be formatted differently, and this affects how we attend to information and what it is like to experience it.

I shall focus on the two distinctions that are based on *representational* formats. One of them, as mentioned, is the difference between analog and digital information. The other one is the related distinction between magnitude-based representations and symbolic, conceptual, and linguistic contents. The goal of examining these distinctions is to explore the claim that visceral valence-structure is unique to consciousness and how valence formats may differ and interface with semantic or conceptual formats. More specifically, while there may be various kinds of phenomenal consciousness, they all share a general type of valence structure, which helps differentiate conscious from unconscious attention. Access to contents need not depend on this valence structure and thus, access consciousness can be conceptualized as distinct from phenomenal consciousness; access consciousness fundamentally depends on attention.

This distinction between valence and symbolic or semantic formats is compatible with diverse types of conscious awareness. One dimension of valence is along the axis of visceral intensity that is characteristic of some experiences. From a phenomenological perspective, intense pain is different from the negative experience we feel when we disagree with someone—one is more intensely felt than the other, although both have negative valence. Some conscious experiences are a lot more visceral and valence-structured than others. There is something overpowering about very visceral experiences, which reveals the crucial role that older parts of the brain play in coordinating neural activation, for instance the regulation of intense fear (LeDoux, 2012). These conscious experiences concentrate all the available attentional resources, not allowing for any degree of distraction. The excessive arousal of extremely visceral states leads to very negative valence. A similar structure is found in positive valence and the regulation of arousal and motivation. We need to live our lives in between the two extremes of excessive arousal and absolute disinterest. This balance articulates preferences and values according to our long-term personal goals and narrative. Homeostatic processes aid at achieving this balance.

Homeostasis helps explain a key aspect of viscerality and valence (Solms, 2021), namely how feeling good is associated with homeostasis—it feels good to be at metabolic equilibrium concerning temperature, blood pressure, and so on. But homeostasis cannot explain all the essential aspects of consciousness, including other formats for non-homeostatically dependent valence. While it is true that we seek to avoid extreme forms of visceral negativity by striving for stability through homeostasis, there is plenty of valence complexity outside homeostatic states, which cannot be reduced to visceral reactions (although see Prinz, 2004). We avoid the vivid pain associated with the loss of physical and emotional homeostasis (e.g., caused by severe injury or extreme anxiety), but this cannot explain a large set of valence structures that are important while we are at homeostatic equilibrium. For instance, a more permanent representation of our values and preferences operates independently of these homeostatic fluctuations. Among these permanent valence-structures, long-term planning based on autobiographic memory and refined preferences and values based on skills and knowledge (rather than homeostasis), play a critical role in providing a more complex valence structure to conscious awareness. They also play an important role in segmenting foreground and background, relating consciousness with attention and integrating various forms of conscious attention within a field of preferences and salient contents (see Watzl, 2017).

Thus, a quite different dimension of valence concerns conceptualized values and preferences structured in terms of autobiographical information. A personal narrative provides a non-visceral and non-navigational perspective. Narratives structure values and preferences according to a ranking of needs and priorities, and they provide access to information that is temporally structured making salient the order of crucial events according to needs and preferences. Narratives are meta-representational because both the visceral and navigational components of awareness are experienced at a moment in time within this complex temporal and preference-based structure. This information then needs further processing in order for needs and priorities to be ranked at a personal or autobiographical, long-term level. These different formats—visceral-valence, navigational-sensorial, conceptual and narrative—provide unique perspectives on conscious experience, and they roughly correlate with the roles of non-cortical and cortical correlates described above. Each can be studied across species, and each plays a distinct role in human conscious awareness. Some species might experience “what it is like” to perceive the world mostly through navigational and visceral formats, while other will have various formats that include visceral valence as well as long term goals and values, imposing a long-term structure to rewards and goals based on attentional salience and inhibition.

Navigation skills are pervasive in nature. A navigational perspective is based on attention skills that concern spatial, temporal, object-based, and feature-based attention, within and across perceptual modalities. These skills constitute perceptual scenes, such as the visual and auditory scenes with their quite distinct features, objects, and geometric structures. As is explained below, egocentric and allocentric frames of reference format various kinds of information in navigational perspectives. Some species rely more on external cues in their navigational perspective while others depend on reliable internal signals. But all must map their egocentric location within a navigational perspective through integrated and cross-modal attention. This navigational allocation of attention varies across species and it determines what it is like for them to move through space (Clark, 2000).

The diversity of conscious perspectives is discussed in more detail below. Here I seek to clarify the meaning of “what it is like” for an organism to experience a content as encoded in a variety of formats. Because of the central role that navigation plays in the behavior of species after the Cambrian explosion (consider predatory behavior), perceptual conscious awareness seems to fundamentally depend on the evolution of spatiotemporal attention across modalities (Haladjian and Montemayor, 2015) and also on quite flexible and general forms of learning (Ginsburg and Jablonka, 2019; Montemayor, 2021). Each sense modality has its own format for representing spatiotemporal relations, which determines what it is like to have perceptual experiences—an aspect of sense-specific perceptual experiences highlighted by Nagel’s (1974) influential article, particularly concerning echolocation, navigation, and proprioception.

Visual perception encodes temporal information differently from auditory perception (Pöppel, 1988) and both integrate information in a three-dimensional spatiotemporal manifold, which is then further integrated into a cross-modal audiovisual field (Callender, 2008; Montemayor, 2013). The spatial, geometric (Wagner, 2006) and topological features of the visual and auditory scenes also need to be integrated into this navigational manifold (e.g., Bregman, 1990; Spence and Squire, 2003). Other sense modalities encode spatiotemporal relations for various cognitive and navigational purposes, in terms of allocentric or egocentric frames regarding the integration of perceptual objects and features. All of the formats for sensorial integration are best understood as magnitude-like, and also as independent from homeostasis, given their metric, rather than strictly “valence” structure (see Montemayor, 2013). They concern magnitudes that can be mapped or scaled, and which represent through approximation, rather than in accordance with precise symbolic systems. These magnitudes have their own kind of compositionality (Montemayor and Balci, 2007) and they involve time, space, number and their metric derivatives such as ratio, speed, or acceleration (Gallistel, 1990). These magnitude-based formats provide a stable way of metrically encoding information from the geometric and topological invariances in the environment, independently of visceral variations in valence—they are somehow “encapsulated” to a substantial degree (Montemayor, 2019a) and operate automatically without much interference from conceptual or visceral-based formats. An issue that must be investigated more carefully is how valence formats based on visceral or homeostatic functions differ from magnitude and analog formats (Maley, 2011; Beck, 2014), and how they might explain various types of conscious awareness, specifically perceptual consciousness.

Issues surrounding the integration of diverse encoding formats played a central role in the development of the psychology of attention. For instance, a key difficulty was to determine if integrating these various magnitude formats requires an object-based (Pylyshyn, 2007) or a feature-based approach (Treisman and Gelade, 1980). Cognitive maps for feature integration into objects underlies perceptual attention, but how this integration occurs at the level of formatting and encoding remains an intricate question (Clark, 2000; Lee, 2021). There clearly is an interface between magnitude formats, core-knowledge, and conceptual formats (Spelke, 1994; Carey, 2009). But an interface of formats is not enough. There is a parsing of formats that provide attentional salience, delineating information into background and foreground, and this partly depends on interest and valence. Yet, different sensory-navigational timeframes must be metrically integrated. Smell, for instance, seems to specify a “background” spatiotemporal encoding while vision and audition seem to be typically at the foreground. But all modalities at the background and foreground constitute a navigational or metrically structured scene. As an illustration, if I am looking for my keys, the conceptual category “key” is at the foreground of my perceptual search, but how I feel at that moment is also determined by information in the background, such as how anxious I am and how urgent it is for me to find my keys. The latter is information with valance that is not fully reducible to the specific conceptual categories that drive perceptual searches.

Besides sensorial formats, other types of conscious awareness that differ in phenomenology, such as imagery, imagination, and memory, also depend on analogous kinds of format-integration. An instance of this type of integration concerned the debate on the continuity of perception with imagery (Kosslyn, 1994; Pylyshyn, 2003). The evidence favors the view that the geometric formatting of the perceptual modalities is continuous with imagery (Kosslyn, 1994). However, none of these perceptual or cognitive formats fundamentally depend on the loss of homeostatic equilibrium and yet, they are all essential in determining what it is like to have perceptual experiences. Some experiences may in fact depend on both, such as emotions and feelings. Emotions are more dependent on homeostasis because they are more “viscerally” processed, while feelings seem to involve judgment as well as categorical representation, or concepts (Damasio, 1999; Feldman Barrett, 2017). Social influence and cooperation, short and large-scale, also shapes the valence and intensity of these mixed formats for emotion and experience, repurposing and refining old areas of the brain into new kinds of formats and connectivity, which is very relevant for the debate on the NCC (see Anderson, 2010).

The key point is that symbolic formats for propositional contents and inferential reasoning are experienced differently from analog and more “iconic” contents, and there is diversity within each type of format. One can illustrate this diversity by comparing the differences in phenomenology concerning different sense modalities. Experiencing visual space is quite different, structurally and phenomenologically, from experiencing auditory space although both are highly iconic or “picture like” and formatted in magnitude or analog terms. This difference also depends on the sensorimotor coupling provided by the sense organs and body, as well as the functions of visual and auditory attention as a kind of action on the environment (O’Regan and Noë, 2001). While the phenomenology of vision and audition are quite different and not exchangeable in terms of qualitative character, perceptual attention is quite flexible, as sensory substitution experiments demonstrate (von Melchner et al., 2000). A plausible interpretation of these findings is that the extrinsic features of perceptual scenes guide perceptual attention across perceptual scenes with quite distinct phenomenology.

Experiencing fear is different from experiencing the urge to buy the latest laptop, although both share some involvement of visceral and arousal signals. The evolutionary purpose and range of intensity of these experiences is also quite different. Falling in love feels very different from planning a trip. In fact, the most interesting experiences have a valence structure that defies planning (e.g., falling in love, laughing with a friend, admiring a full moon). These are experiences that involve an effortless type of conscious attention, one in which the experience of the self seems to vanish (Csikszentmihalyi, 1997). The point of these examples is that although falling in love and planning a trip depend on our preferences, the way we experience them is encoded very differently (i.e., we cannot really “plan” to fall in love in spite of the fact that how we fall in love reflects somehow our preferences). Thus, these examples make a distinction between formats, rather than explain more specifically their positive characterization, which is beyond the scope of this article.

Thus, a vast variety of experiences have valence, but they do so in very different ways. An issue that deserves more investigation is, which forms of valence are more primitive or biologically fundamental than others? One option is that consciousness provides a uniform type of valence across various types of experience. A more pluralistic approach, like the one endorsed here, would deny this claim by adding other types of valence that are independent from homeostasis or biological arousal. But these views need not be antagonistic, since there might be something universally related to visceral or homeostatic processing concerning what it is like to have any experience, even if other formats are also fundamentally involved.

This analysis of the diversity of conscious awareness in terms of formats, as well as their possible NCC, leads to the following three insights:

#### First Insight—There Are Epistemic Differences Among Formats and Sense Modalities

Some experiences are formatted in such a way that their role is more semantic and epistemic than other experiences—more associated with accurate access to information than other experiences. This could occur implicitly as in cases in which one has a feeling that something is wrong or messy but cannot articulate exactly what information grounds this judgment. Phenomenologist have given vivid examples of this phenomenon (e.g., Merleau-Ponty), and neuroscientists have shown that such implicit states are relevant to explain the behavior of patients with brain lesions (Bartolomeo and Dalla Barba, 2002). By having a conceptual structure or format, such implicit states are more readily accessible to explicit judgment. For instance, by having a conceptual formatting, some visual and auditory experiences play essential epistemic roles concerning justification, evidence, and perceptual knowledge. It is likely that the inferential structure and the epistemic or semantic roles of experiences are grounded in attention as a kind of cognitive and *epistemic agency* (Fairweather and Montemayor, 2017; Montemayor, 2019b). In other words, attention can be a form of mental action (Wu, 2014) under the control of the agent that satisfies representational and cognitive needs. If so, these experiences require essentially the involvement of attention routines. Many of these forms of access based on attention may require conceptual or symbolic (“language-like”) formats.

A magnitude-based format can encode information iconically or through picture-like and map-like encodings or representations (e.g., Fodor, 2008; Echeverri, 2017). These iconic formats can provide navigational knowledge and non-conceptual or pre-symbolic forms of experiencing on the basis of homeostatic and visceral states, including the vivacity of color, pitch, acidity, acridity, heat, and other magnitude or intensity-based contents with a specific sense-modality format. Non-human species must have experiences formatted in these ways, perhaps a variety of them. Experiencing what it is like to navigate an acrid/fragrant environment differs drastically from navigating a landscape of sounds coming at different speeds. All species depend on such sense-specific types of navigational capacities. There is no monolithic “what it is like” that unifies all these formats. This, however, is compatible with the claim that there is a unity to awareness and the first person point of view, as clarified in the following section.

#### Second Insight—There Are Sense-Modality Differences Based on Informational Foreground and Background

Human consciousness foregrounds visual experiences above all others. Many of our linguistic expressions concerning knowledge and understanding are visual, such as “can you see my point?”, “seeing is believing,” or “I understand their view” among many others. We place great emphasis on visualization in our efforts to understand and model the world, in science and in general. Although typically audiovisual experiences are highly integrated, vision has dominated the discussion on conscious awareness (most examples in philosophy concern color or the features of visual objects). Attention research has also focused on the epistemic functions of vision, such as searching, tracking, interpreting, classifying, generating a statistically significant “gist” of information, and the unlimited forms of learning based on visual classification and categorization. This emphasis on vision has “epistemicized” our understanding of conscious awareness.

Given that many forms of visual attention routines are fundamental for all these epistemic tasks, research on consciousness, particularly in philosophy, may have characterized the overall character of conscious awareness in a biased manner, by emphasizing mostly the epistemic aspects of vision. At the same time, this marked emphasis on epistemic functions may have produced a misunderstanding concerning the conceivability scenarios used to establish the non-functional character of conscious awareness (e.g., philosophical zombies, Mary). Part of this misunderstanding is that qualia are typically associated with some kind of visceral reaction that is not reducible to epistemic functions. But even in vision, color experiences have a salient visceral component, which cannot be simply described as epistemic or categorically-based (Humphrey, 2006, 2011; Haladjian and Montemayor, 2015) and which has older neural paths in the brain (Pauers et al., 2012).

Moreover, the centrality of vision in epistemology does not entail the cognitive priority of vision over the other sense modalities, since the other modalities play a critical epistemic role in many other species that have lived in our planet for much longer than us, and for whom olfaction and audition are more salient. Non-visual modes of experiencing the world provide information that escapes visual cognition, and which are capable of reshaping visual information. Since, for humans at least, visual experiences typically receive more attention resources, the key difference between vision and the other modalities concerns salience. As mentioned, attention plays a critical role in this process of delineating attentional foreground and background. But what is novel about understanding these differences among the senses in terms of both format and salience is that the senses can be classified according to two axes corresponding to their functional roles: *their salience and their integrative roles*. Relations among the senses modulate attention and interconnect the different phenomenal characters of each sense. For instance, visual awareness can be influenced by acoustic sensations (Kusnir et al., 2011) and in brain-damaged patients acoustic input can decrease visual neglect (Robertson et al., 1998).

Some senses play highly integrative roles in spite of being generally in the background, such as proprioception and olfaction. For example, smell is more viscerally experienced and is determined more by valence than vision (a typical claim about smell). It is also more integrative and unifying as a background or “atmospheric” sense. More precisely, smell provides *familiarity* and a kind of “atmospheric unity” to conscious awareness, with strong connections to the valence of autobiographical memory and emotions. Some senses are, accordingly, more atmospheric (for lack of a better word) and familiarizing than others. They are based on a visceral kind of familiarity that is not purely epistemic, semantic or categorical—they provide ambience rather than merely judgments and justifications (the visceral components of color likely play a similar role in vision). There is a kind of informational “speed” vs. experiential “depth” characteristic of the phenomenology of different senses, along the axes of salience and integration. When one plays hockey or soccer, visual and auditory features integrate seamlessly and rapidly into the audiovisual scene, allowing us to quickly react to stimuli that are expeditiously and automatically processed. Yet a strong smell, even in the middle of a challenging game, can strongly evoke a personal memory, either pleasant or unpleasant. This more visceral experience at the “background” of our cognition seems to be a uniquely important feature of our overall phenomenology.

Low salience provides integration and familiarity. Cases in which it is difficult to articulate the specific contents that one is experiencing, but which permeate a perceptual scene such as a general sense of discomfort, anxiety, or uncanniness, illustrate this type of low but general salience. For this reason, the familiarity of conscious experience depends at least partly on this kind of low salience. In demanding scenarios, accessible contents are highly salient in order to make possible skillful performance. Thus, “low” and “high” kinds of integration and salience frame attention and awareness. An example of a “low-low” scenario is when we are waking up or recovering from anesthesia and feel our body’s position without knowing where we are, feeling some overall sense of uncertainty. We are aware of these experiences as a general “mood” without any specific content being salient. A “high-high” situation is when we are talking online, responding to questions and tracking who is asking them. Here the visual and auditory contents are the most salient, and yet sudden changes in smell or proprioception would quickly enter awareness from the background.

Each sense has a unique topology, geometry, semantic structure, and a phenomenology-based similarity metric that determines what it is like to experience contents from within the formatting and valence structure of a specific sense modality (see Lee, 2021 for discussion). The fact that smell and taste implicitly shape our experience of the world does not mean that they are phenomenally unconscious—the point is that we rarely *access* or notice them unless they become viscerally dominant and foregrounded. A question that deserves further investigation is to what extent attention without consciousness could play the most salient epistemic roles of cognitively integrated information. Based on the evidence, it seems that even if attention could play *all* of the epistemic and semantic roles of cognition, consciousness would still be essential to provide familiarity and valence to experience, both magnitude and visceral-based.

The difference between epistemic role and emotional valence has a further philosophical implication concerning the value of consciousness. Epistemic value, associated with the quality of evidence, belief justification, and the capacity to acquire knowledge differs from, and on occasion may clash with, moral value. If attention suffices to explain most of our cognitive epistemic functions (Fairweather and Montemayor, 2017), then consciousness may add a moral and aesthetic dimension to our cognitive lives. Two kinds of motivation are at play here. Epistemic motivation concerns the satisfaction of representational and cognitive needs concerning knowledge acquisition. Moral, aesthetic, and social motivation, while typically related to epistemic motivation, concerns arousal and the satisfaction of empathic and emotional needs. If this is the case, then consciousness would be the source of a deeper sense of “belonging” and familiarity, associated with a life worth living (Humphrey, 2011). This is an intricate issue, since consciousness might be the source of various types of value (Kriegel, 2019). An advantage of the dissociation between consciousness and attention (Montemayor and Haladjian, 2015; Jennings, 2020) is that it allows us to tackle this topic by examining epistemic function in terms of attention, including cross-modal attention and inferentially integrated attention, as well as so-called “access consciousness.” By contrast, consciousness or more precisely information that is unique to conscious processing could be studied by appealing to valence, homeostatic function, and various navigational formats for experiencing contents.

#### Third Insight—An Invariance in the Metric and Magnitude Structure of Perceptual Scenes Is Compatible With a Radical Diversity in Phenomenology

Non-human species navigate the same world we perceive, and can coordinate actions in large groups, across space and time. However, the fact that attention skills allow us and animals to successfully navigate our environment by preserving spatiotemporal, causal, and other metric invariances, does not entail that we all experience it in the same way. To the contrary, while joint attention and spatial attention depend on such invariances, the specific valence structure of each sense modality may vary significantly across species. Here again, a key constraint for studying this multiplicity is the interplay between consciousness and attention and more specifically, the precise difference between attentional and conscious *information processing*. As mentioned, sensory substitution illustrates that the same contents can be processed across senses, in spite of drastic differences in phenomenology.

With respect to the unity of conscious awareness, sensorial consciousness integrates spatial, temporal, and statistically relevant features of the environment in an overall unified conscious space or “field” of awareness, with its own unique geometric and topological features. Navigation imposes the further constraint that allocentric information must be integrated and updated with egocentric information constantly, and in an “indexical” fashion (e.g., determined by the cognitive equivalents of the linguistic expressions “here,” “now,” and “I”). Studies confirm that certain temporal patterns relevant for the integration of stimuli determine entry into awareness, for instance the presence of awareness negativity and later prefrontal activation (Dembski et al., 2021). This activation threshold may be a necessary component of consciousness, besides thalamic and non-cortical activations, as explained above. The key point for present purposes is that such integration constitutes a *navigational perspective* that may not be sufficient to ground a full first-person perspective, as is explained further in the next section, which critically examines extant accounts of the first-person perspective.

## A Diversity of Perspectives and the Unity of the Self

So far, the general argument of this article has relied on the fact that consciousness depends on attention, and that this entails the diversity of consciousness. The argument, in various forms, concludes that since there are many types of attention with different functional and formatting aspects, the dependence of consciousness on attention entails similarly diverse types of conscious experiences. Two categories were proposed in order to organize this diversity of attention in terms of two kinds of agency. One kind of agency is largely epistemic, conceived broadly as a set of unified attentional capacities that satisfy representational, cognitive, and rational needs. In the literature, this kind of attentional agency roughly resembles access consciousness, and these notions may actually be identical (Stoljar, 2019). The other kind of agency is based on skills that are formatted in accordance to magnitude and valence encodings. These visceral and analog formats provide a biologically rooted and bodily engaged phenomenology, which seems to be unique to conscious information processing. The difference between epistemic or knowledge-based skills and the more visceral or valence-based skills can be examined in terms of differences between consciousness and attention. Consciousness is based on attention and both play critical and mutually supportive cognitive roles, but it is useful, for theoretical and experimental reasons, to distinguish them. What seems unjustified, given the empirical evidence, is to assume that consciousness is a single type of “unity” given the plurality of attention skills.

The focus now is on the conscious perspective of an organism, rather than the more generic notion of agency defined as a set of cognitive and motivational capacities to achieve goals, broadly construed. More precisely, the first person perspective, which is supposed to be a unique and primitive feature of phenomenal consciousness, differs substantially from the merely navigational perspective described above, in at least two key respects. First, the conscious perspective of a human does not seem to be sufficiently captured by a set of capacities formatted in diverse ways because language, introspective and overt, plays a fundamental role in the consolidation of such a reflective perspective. Second, the kind of memory required for the richly autobiographical conscious perspective of a typical human being cannot be captured by a navigational and “moment-to-moment” perspective (for discussion see Hoerl and McCormack, 2018; Montemayor, 2019c). Thus, the main focus of this section is “the self ” (understood in terms of the first person perspective), as a source of unity in cognition for both consciousness and attention. This unity, however, is compatible with a diversity of attention types, as well as with a correlative diversity of kinds of conscious awareness.

The locution “first-person point of view” has played a pivotal role in the debate about the nature of consciousness. However, it is not entirely clear from the extensive literature on this issue just how rich this perspective should be in order for it to count as a *conscious* perspective. This section argues that there is plenty of diversity here as well and that this diversity can also be categorized in terms of the difference between attentional and conscious information processing. What it is like to be an organism from a first-person perspective has been one the main considerations behind all arguments for the non-reducible nature of conscious information to scientific, functional, or “third person” information (Nagel, 1974; Chalmers, 1996). But some of the navigational “self-locating” perspectives are best understood in terms of attention, rather than consciousness. These perspectives are not as robust as a human’s first person perspective but they provide unity by being egocentric in two ways: in terms of a point of access in a navigational framework centered on the individual (based on attention skills) and also as the viscerally felt and emotionally engaged perspective of a biological organism. Therefore, the unity afforded by the first person perspective is compatible with diverse forms of attention and consciousness, particularly concerning self-locating types of information that are a condition for the possibility of the first person perspective.

This approach based on the distinction between consciousness and attention favors the scientific methodology promoted by Dennett (2005). According to this methodology, *a priori* methods should be complemented, and ideally replaced, by empirical findings. An *a priori* approach is assumed in a good portion of the contemporary philosophical accounts of consciousness, including the influential hard problem. The basic intuitions about “zombies” underlying the hard problem have been contested as unreliable (e.g., Fischer and Sytsma, 2021), so we have justification to give at least equal weight to scientific findings and to avoid relying exclusively on intuitions about consciousness (see also Melloni et al., 2021). However, contra Dennett, I argue that there is no unique or single narration-based perspective that creates the “illusion” of privileged access. Rather, there are multiple perspectives, all of which can count as a conscious “what it is like” to be an organism. While this claim is not entirely incompatible with Dennett’s (1991) “multiple drafts” theory, it shows that the variety of conscious perspectives needs to be studied in more detail and also that the narrative perspective of human consciousness need not be merely an illusion or a “bag of tricks, ” but a more fundamental characteristic of having language and complex memory capacities as the basic framework for valence and access.

Agents have capacities or competences that allow them to satisfy multiple needs. Navigational perspectives unify various capacities in terms of a diversity of formats for access and conscious valence. Most species rely on some kind of navigational perspective, regardless of how rudimentary it might be. Such a navigational perspective can be explained in terms of integrated attention skills. As explained before, different quality and categorical metrics (similarity or conceptual) vary across magnitude mappings at the cross-sensorial level. The navigational and perceptual perspective of an organism is not as rich as a narrative-based and linguistically formatted perspective, but it certainly is a way of experiencing “what it is like” to be an organism.

Human conscious awareness is heavily influenced by language and its semantic format. It is also “drafty” or open to interpretation (Dennett, 1991) thereby creating a narrative structure for the first-person perspective. Language is experienced in a variety of ways (e.g., as intended social communication, as public commitment, as inner speech and thought). We experience truth in language, as well as deception. Long-term planning in humans typically requires a narrative structure that serves as the basis for value and preference rankings. As it has been argued at length in the literature, such an internal perspective is not reducible to knowledge of facts, presumably, even in the absence of linguistic skills (Nagel, 1974)—although it is very hard to conceive of a first-person perspective without any communicational skills. Episodic memory provides an egocentric perspective on memory—a kind of self-knowledge dependent on the past that frames and makes possible long-term planning (Tulving, 1985). This perspective is essential in decision making, and value rankings based on the visceral and emotional valence-structure of awareness may be the only aspects of this perspective that are uniquely conscious. Other components of episodic memory may be understood in terms of attention without awareness (Montemayor, 2018). Thus, linguistically framed memory provides its own way of framing valence and content, long- and short-term.

Some authors emphasize a very basic navigational and visceral perspective as the fundamental characteristic of consciousness (Merker, 2007; and this is a plausible interpretation of Nagel, 1974). According to this view, associated with non-cortical theories, the richer and more semantically structured long-term memory could be largely unconscious, since many of these more complex cognitive functions can be fully understood in terms of attention. A radically different account emphasizes language and the narrative aspects of episodic memory as key ingredients of the first-person perspective (Dennett, 1991; Rudder-Baker, 2013), which determines what it is like to be a conscious organism with that specific perspective. To motivate the issue of diversity here, in the context of defining the requisites of a necessarily conscious first-person perspective, consider the variety of perspectives that may exist between raw visceral awareness and long-term planning based on autobiographical memory.

In the phenomenological tradition, a variety of perspectives have always been discussed as central aspects of the examination of consciousness, rather than an emphasis on a single notion of “first-person perspective.” There is, for example, the conception of consciousness as a unified *stream* of contents, framed by a non-egological, temporal, and compositional “parthood-based” structure (Dainton, 2000). A more reflective sense of self, or a narrative-self along the lines described by Dennett’s “multiple drafts” theory necessitates long term memory, attention to context, and fundamentally, linguistic skills. Thus, there is a variety of conscious perspectives discussed in the literature, ranging from a viscerally structured streams of consciousness, deeply related to the phenomenology of time, particularly as developed by Edmund Husserl (see Zahavi, 2005; for an examination of the temporally metric perspective for sense specific and cross-modal contents, based on findings on time perception, see Montemayor, 2013) to fully articulate, linguistically structured, narrative accounts. In fact, Neisser (1988) distinguishes between several “first person perspectives,” namely ecological, interpersonal, extended, private, and conceptual notions of the self. Other authors postulate even more notions of the self (see Strawson, 1999).

Here we find an interesting, and perhaps unique, symmetry between the varieties of perspectives discussed in the literature on the phenomenology of experience and the varieties of attention, understood as an epistemically agential capacity. For instance, Ganeri (2017) proposes a non-egological account of the phenomenology of the self that is nonetheless, at least in principle, compatible with a diversity of attention capacities unified into a subject-level *epistemic agency* (Fairweather and Montemayor, 2019). As mentioned, the egocentric frame of navigation in all species with locomotive capacities can be conceived as a set of attention skills for spatiotemporal cognition, and the capacities for integrating stimuli into perceptual features and objects can also be conceived as a kind of perceptual attention-perspective.

Language, as a set of epistemic skills that require syntax, semantic, and pragmatic reasoning, has almost dropped from discussions concerning the necessary conditions for conscious awareness, partly because this delineation of consciousness would completely exclude the possibility of consciousness in other animals. But the thesis that language is necessary for conscious awareness has a long tradition in the history of Western philosophy, playing a critical role in the Cartesian and Kantian conceptions of the mind. More recent approaches placed a great deal of emphasis on linguistic capacities as fundamental to the development of consciousness (Jaynes, 1976; Dennett, 1991). From an empirical point of view, however, this view would go against evidence concerning conscious but not explicit or accessible content, patients who have awareness independent of language, and also many cases of awareness in other species, for example monkeys (Cowey and Stoerig, 1995).

Rudder-Baker (2013) delves more deeply than most authors into the view that language is a necessary condition for a robust first-person perspective, constitutive of human conscious awareness (although she does not discuss consciousness in depth). She critically assesses the work of Metzinger (2003), and also argues against Dennett’s “illusionist” naturalism. Her aim is to propose a robust metaphysical defense of the self on the basis of concepts and language, compatible with scientific inquiry and methodology. Her work is relevant to the present discussion for two reasons. First, she provides a full explanation of how scientific evidence may be compatible with the rather mysterious notion of a “self ” understood as a first-person point of view irreducible to third-person explanations. Second, her account of the first-person perspective is also fully compatible with a diversity of types of consciousness and attention.

In particular, on Rudder-Baker’s view, there are two varieties of “personal indexical, ” or the mental equivalent of the linguistic expression “I.” These two senses of “self ” can be roughly characterized as conscious-navigational (or even visceral) and access-attentive, based on linguistic skills and conceptual inference. Baker argues against Metzinger precisely on this basis, because she claims that Metzinger’s account confounds these two senses of self. On Metzinger’s account, according to Rudder-Baker, “I” could be “the whole information-processing system” but it could also mean “I*” which refers to the “self-model” (Rudder-Baker, 2013, p. 96; the terms I and I* are Baker’s). Rudder-Baker argues that, necessarily, one has a robust first-person perspective only if one has a “self-concept,” which fundamentally depends on a battery of empirical concepts whose content is determined in *public language*. This implies, besides the dependence on language, that social relations and conceptualized social interactions are also fundamental to have a robust (rather than merely navigational, as in animals) first-person point of view (p. 137). Since a robust perspective requires access to concepts and joint attention to socially structured communication, this robust perspective is best understood in attentional, rather than merely qualitatively conscious (and solipsistic) terms. Thus, Rudder-Baker’s view entails a fundamental difference between navigational and robust first-person perspectives, correlative to the distinction between consciousness and attention and the dependence of consciousness on attention.

Even if Rudder-Baker’s account is mistaken, for instance, because of its strong assumptions concerning the necessity of concepts and language, the distinction between two types of perspective, one navigational and the other access-attentive, adequately captures what is distinctive about the perspective of social organisms with larger brains, such as humans. To clarify, the navigational perspective is certainly attentive, in the sense that it involves an epistemic agent’s capacities to attend to stimuli, which allow the agent to acquire knowledge about the environment. But this navigational perspective need not be self-referential, conceptual, or even “accessible” through concepts or language. This basic navigational perspective could be deeply related, and even necessarily, to visceral valence and analog metric formats. Unlike the more robust, self-referential perspective, the navigational perspective might be essentially conscious (in sharp contrast to Dennett’s and presumably Rudder-Baker’s views).

The treatment of diverse forms of consciousness and attention according to their format illuminates this distinction between two perspectives. Diverse types of agencies in different formats evolved independently, and became highly unified into a perspective for either navigation or self-reference. One perspective is tied to sensorial stimuli while the other expands in time through counterfactual inference and narrative. These two kinds of indexical or self-referential perspectives provide a type of self-knowledge (content *de se*, not reducible to contents about references or propositions; see Perry, 1979). This variety of perspectives implies different forms of *information processing*. We need a comprehensive examination of how information processing relates to attention and consciousness as information is integrated into these two distinct kinds of perspective in order to determine the nature of the dependence of consciousness on attention.

The role of language in the integration of a first-person perspective also deserves further exploration. Even on an “illusionist” account in which consciousness is not a primitive and irreducible datum concerning “what it is like to be an organism,” consciousness still has some useful purposes. Dennett’s favorite picture of consciousness is a “thought bubble” with a stream of consciousness, linguistically formatted (a Saul Steinberg illustration for *The New Yorker*). Such a conscious perspective is intrinsically “chatty,” overtly and introspectively, and it may be unique to humans, given that the full range of language skills has not been identified in other species. Conditions that depend on language function and narrative formation, such as verbal hallucinations and a loss of familiarization with the self, such as schizophrenia and depersonalization disorder, may shed light into the role of language in integrating a robust and autobiographical first-person perspective.

## Methodology and Diversity: The Armchair and the Lab

To conclude, it is worth highlighting the importance of examining the diversity of kinds of consciousness as a matter of scientific methodology. The implications of the argument above for methodological practices are straightforward. Consciousness depends on attention for information processing purposes. Since there are multiple kinds of attention, in different formats and assembled as diverse kinds of intelligent capacities for agency, this entails a diversity of conscious information. Even if one examines the unity of consciousness through the first-person perspective, one can distinguish at least two kinds of perspectives. Various relations between consciousness and attention (Montemayor and Haladjian, 2015) can expand the diversity of conscious information and allow for a more detailed examination of animal consciousness according to diverse evolutionary paths (see Edelman, 2003 for how this diversity can be integrated in accordance to evolutionary principles; see Haladjian and Montemayor, 2015 for an evolutionary account that centers on consciousness and attention). Therefore, multiple methodologies must be used to explore what makes information uniquely conscious, what is exactly the relation between diverse forms of attention and diverse forms of consciousness, and what is the relation between conscious information and diverse forms of intelligence.

Methodological diversity is already present in the contemporary literature, as the discussion above on the NCC demonstrates. Even the methods for defining consciousness reflect theoretical diversity. The Integrated Information Theory (Tononi et al., 2016), for instance, addresses specifically the relation between consciousness and information, but proceeds axiomatically and *a priori*, in order to define consciousness deductively from first premises (see Montemayor et al., 2019, for criticism). Most theories proceed *a posteriori*, or by experimental method, using imaging, reports, and behavioral data in order to identify the NCC or neural signatures of conscious information. A renewed empirical perspective should include animal cognition and information theory. Such a methodological approach should also explain the dependence of consciousness on attention and examine possible degrees of dissociation between them. Not all extant theories can be right, of course, but no unique approach should be favored above others either—all theories might provide important insights, even within a single study. A conciliatory methodology to study consciousness with an emphasis on empirical methods, and with a more explicit focus on attention and information, deserves more development and support.

This diversity-based approach also allows for new philosophical interpretations of classic thought experiments in the literature that rely on “armchair” intuitive methods. For example, according to philosophical lore, after a lifelong career as the world’s authority on color research, “black-and-white” Mary finally experiences red for the first time. Mary’s situation, introduced by Frank Jackson (1982) in his influential article, is one of the most discussed thought experiments in the literature on consciousness, and has been interpreted in multiple ways. Among them we find that the thought experiment: (i) is based on unjustified intuitions or that it confounds different definitions of knowledge; (ii) shows something interesting about concepts, but not about reality; and (iii) presents a radical challenge to physicalism. Mary’s alleged new knowledge has been described in terms of an informational puzzle that requires a distinction between descriptive and practical knowledge (Lewis, 1988), the semantic features of indexicals (Stalnaker, 2008), or a positive reassessment of dualism, including panpsychism (Chalmers, 1996). It is assumed that all the interpretative options have been exhausted. In fact, the main views about the nature of consciousness are presented in terms of the change Mary undergoes after experiencing red for the first time, thereby *delineating* the major boundaries of the debate between physicalists and non-physicalists.

But if what is said above is correct, something fundamental has been ignored in all these accounts of Mary’s situation. Perhaps the territory has not been thoroughly charted—attention needs to enter the picture. Mary’s case needs to be interpreted in terms of *joint attention* for communication, and particularly, in terms of how the public nature of communication bears on the nature of phenomenal consciousness. Here is an interesting possibility: either there is nothing special about Mary’s new situation with respect to information or her situation must be understood in *normative* terms—the distinction is not merely one of information “type,” defined as new or old knowledge, but rather, *as a difference in value or valence structure*. Accepting the second option is very plausible, and based on a diverse approach, it provides a fresh formulation of why we think consciousness is so hard to explain. Understanding Mary’s situation in terms of joint attention and consciousness explicitly contrasts the publically constrained conceptual capacities and linguistic tasks that Mary is fully competent in performing (concerning color recognition) with the new information she learns about color, which is supposed to be deeply private. Although her new capacities are also attentional and inferential, they are not referentially or epistemically constrained through public language and joint attention.

Rarely has Mary’s predicament been interpreted based on the “value intuition,” and most interpretations are based on the knowledge intuition. The problem with the knowledge intuition is that knowledge requires achievements that are *publically assessable*, while the intrinsic value condition may be independent from any publically assessable knowledge-based criterion. A value-based solution states that what Mary learns has emotional and aesthetic value, and that this is the main explanation of our intuition that what she learns is profoundly and personally significant (Humphrey, 2011). Even though she is perfectly competent as an *epistemic* agent, she cannot articulate what she learned in terms of the epistemic constraints of joint attention. She gains, more specifically, *a visceral and atmospheric way of relating to color—the intrinsic value of qualia*. Thus, the root of our intuitions on Mary’s situation concerns two independent sources of normativity and value (epistemic and aesthetic), rather than two independent sources of knowledge.

Mapping the mind is one of the most fundamental scientific challenges of our time. The relation between consciousness, attention, and intelligence is central to this task. A diverse approach to consciousness may produce new insights for neuroscience, psychology, and philosophy. I have not discussed metaphysical views of consciousness in any detail, such as panpsychism, because my goal is to clarify the extant evidence on the neuroscience and psychology of consciousness. Panpsychism is a controversial view of the nature of consciousness and the universe. But even if one adopts such a view, here too, a diverse perspective could help advance the debate concerning how physical reality relates to the conscious mind. This is a very intricate issue, but I shall conclude by merely remarking that diverse approaches already inform the interpretation of quantum mechanics—there is no single interpretation adopted universally and a variety of perspectives frame the debate on the basic structure of reality, which also offer a variety of perspectives of how the mind relates to the world at large (see de Barros and Montemayor, 2019).

## Data Availability Statement

The original contributions presented in the study are included in the article, further inquiries can be directed to the corresponding author.

## Author Contributions

The author confirms being the sole contributor of this work and has approved it for publication.

## Conflict of Interest

The author declares that the research was conducted in the absence of any commercial or financial relationships that could be construed as a potential conflict of interest.

## Publisher’s Note

All claims expressed in this article are solely those of the authors and do not necessarily represent those of their affiliated organizations, or those of the publisher, the editors and the reviewers. Any product that may be evaluated in this article, or claim that may be made by its manufacturer, is not guaranteed or endorsed by the publisher.

## Footnotes

^1^I am grateful to an anonymous Reviewer for helping me clarify why these distinctions neither solve nor are relevant to address the diversity problem discussed in this article.

^2^I am grateful to an anonymous Reviewer for suggesting this possible source of phenomenal diversity grounded on attention.
